# Do simple screening statistical tools help to detect reporting bias?

**DOI:** 10.1186/2110-5820-3-29

**Published:** 2013-09-02

**Authors:** Romain Pirracchio, Matthieu Resche-Rigon, Sylvie Chevret, Didier Journois

**Affiliations:** 1 Department of Anesthesiology and Critical Care Medicine, Hôpital Européen Georges Pompidou, Université Paris-Descartes Sorbonne Paris Cité, Paris, France; 2 Department of Informatics and Biostatistics, Unité Inserm UMR S717, Hôpital Saint Louis, Université Paris 7 Diderot, Paris, France

**Keywords:** Reporting bias, Reporting, CONSORT, Fraud

## Abstract

**Background:**

As a result of reporting bias, or frauds, false or misunderstood findings may represent the majority of published research claims. This article provides simple methods that might help to appraise the quality of the reporting of randomized, controlled trials (RCT).

**Methods:**

This evaluation roadmap proposed herein relies on four steps: evaluation of the distribution of the reported variables; evaluation of the distribution of the reported *p* values; data simulation using parametric bootstrap and explicit computation of the *p* values. Such an approach was illustrated using published data from a retracted RCT comparing a hydroxyethyl starch versus albumin-based priming for cardiopulmonary bypass.

**Results:**

Despite obvious nonnormal distributions, several variables are presented as if they were normally distributed. The set of 16 *p* values testing for differences in baseline characteristics across randomized groups did not follow a Uniform distribution on [0,1] (*p* = 0.045). The *p* values obtained by explicit computations were different from the results reported by the authors for the two following variables: urine output at 5 hours (calculated *p* value < 10^-6^, reported *p* ≥ 0.05); packed red blood cells (PRBC) during surgery (calculated *p* value = 0.08; reported *p* < 0.05). Finally, parametric bootstrap found *p* value > 0.05 in only 5 of the 10,000 simulated datasets concerning urine output 5 hours after surgery. Concerning PRBC transfused during surgery, parametric bootstrap showed that only the corresponding *p* value had less than a 50% chance to be inferior to 0.05 (3,920/10,000, *p* value < 0.05).

**Conclusions:**

Such simple evaluation methods might offer some *warning signals*. However, it should be emphasized that such methods do not allow concluding to the presence of error or fraud but should rather be used to justify asking for an access to the raw data.

## Background

In a world where medicine is supposed to be based on evidence, the question of how evidence-based the published results should be appraised and translated into clinical practice is of crucial importance [1,2]. To facilitate the appraisal, important efforts have been made to propose reporting checklists, trials registries, and to extend conflict-of-interest disclosure. The CONSORT [3] statements has undoubtedly helped to improve the reporting of clinical trials. The requirement of a registration number helps the reader to verify that the methodology and the endpoints have not changed after completing patients’ enrolment [4,5]. Moreover, many journals now ask for professional statisticians to review the manuscripts [6]. Despite such efforts, the high prevalence of reporting bias as well as several examples of high-grade research findings published in major medical journals, but refuted by subsequent evidences, maintain doubts around evidence-based medicine [7,8].

In an ideal world, major scientific journals should ask the researchers to provide their raw data to allow an external verification of the results [8]. However, while such policies are lacking, it is currently difficult to verify the accuracy of the published data. It is the editors, reviewers, and readers’ responsibility to appraise the research reports, before translating the results into clinical practice. In this context, we proposed some simple statistical screening tools that could help to detect certain types of reporting bias [9]. Our objective was to test whether such simple tools applied to a manuscript known to be fraudulent [10,11] would have helped to detect some *warning signals* of poor quality. Such warning signals would have justified asking the trialists for more detailed information concerning the raw data.

## Methods

We first provide a concise description of the methods previously proposed [9]: evaluation of the distribution of the reported variables; evaluation of the distribution of the reported *p* values; parametric bootstrapping and explicit computation of the *p* values.

### Variable distribution

In many papers, data are reported as if they were normally distributed, while usually, the authors only assume that the data are normally distributed. *First*, the use of summary statistics, such as means and standard deviations, might not adequately reflect the distribution of a nonsymmetrically distributed variable. Although rarely followed, the CONSORT statements recommend using mean and standard deviations (eventually associated to the interquartile range) for symmetrically distributed variables, and median with its interquartile range for other variables [3]. Worse still, parametric statistical tests, such as the Student *t* tests or Analysis of Variance (ANOVA) often are inappropriately used [12]. Such tests might be inappropriate to analyze nonnormally distributed or skewed data, especially when the sample size is not large enough. In such situations, alternatives might either rely on using nonparametric statistical tests or only on comparing the confidence intervals without any statistical tests [13]. However, the fact that a variable is nonnormally distributed does not necessarily imply that the result of a parametric test is invalid. *T* test, for instance, is quite robust to nonnormal data providing sample size is not small and the data are not too skewed. It also should be emphasized that several transforming functions have been proposed to convert non-Gaussian distributions to gaussian form, to eliminate or substantially reduce non-Gaussian characteristics of positive skewness and peakedness [13].

Hence, it is of interest to analyze the distribution of reported variables. *First*, the reader should question whether a given variable could intrinsically behave normally or not. For example, duration variables (length of stay in hospital or a length of mechanical ventilation, for instance) are usually not normally distributed [12]. Because duration cannot be negative, but can trend toward infinity, its distribution is usually asymmetric. *Second*, the reported summary statistics can help the reader to refute the assumption of normality, for example, when a strictly positive variable has a standard deviation close to or even larger than its mean. This means that the variable distribution is wide, but also that, given the fact that negative values are impossible, its distribution is likely to be asymmetric. *Third*, critically looking at the summary statistics can help the reader to detect some lack of variability in the data, which can be related to data smoothing for instance. This point can easily be checked by looking at the variability reported in the literature for similar measures.

### Baseline covariate distribution between groups

Statistical testing should be avoided when evaluating covariate balance, because usual tests are not designed to accept the null hypothesis. However, most published manuscripts report such statistical tests. Analyzing the results of such statistical tests could help to detect poor quality data.

#### P values distribution

If the randomization was adequately performed, baseline characteristics distribution should be balanced between the two randomized groups. Under such a null hypothesis (i.e., the two groups have similar baseline characteristics), the *p* values referring to the comparisons of baseline independent characteristics should follow an Uniform distribution over the interval [0,1] [14]. In other words, the *p* values should be uniformly distributed between 0 and 1. In case of fraud, the authors are usually tented to produce *p* values all close to 1. Hence, when *p* values are all close to 1, one should probably consider this result as a warning signal. As an example, we propose to distinguish a two-class partition of the *p* values (<0.5, ≥0.5), to check graphically the observed distribution of the reported *p* values in these two classes and to see if it is compatible with Uniform distribution. Of course, this evaluation relies on the fact that the authors reported all baseline characteristics that they compared. If they chose not to report some of the baseline covariates, such an adequacy test to Uniform distribution could not be adequately performed on the subset of reported *p* values due to missing—and potentially informative—*p* values.

#### Explicit p value computations

Based on the reported summary statistics (means and standard deviations for instance), one also could compute the *p* value of a Student *t* test (formula provided in Additional file 1). The *p* values for Fisher and Chi-square tests also can be computed retrospectively from the data reported in a contingency table. Moreover, it should be emphasized here that *p* values are probabilities that are use to reject or not the null hypothesis according to some prespecified rejection rules. Multiplying statistical tests intrinsically inflates the type I error, i.e., the probability to reject the null while in fact it is the truth. To address this risk, one should adapt the prespecified rejection rules—that is adjust the *p* value threshold for statistical significance [15].

### Parametric bootstrap

The parametric bootstrap [16] is a simulation procedure that consists in randomly generating many independent datasets on the basis of the reported characteristics of the population and some knowledge about the distribution law. Simulations allow one to recreate a large number of virtual datasets retrospectively. Simulating a dataset requires: 1) a location parameter (mean or median for example); 2) a dispersion parameter (standard deviation or 95% confidence intervals for example); and 3) some knowledge about the distribution law. The parametric bootstrap consists in randomly generating N independent datasets of size *n* on the basis of the reported sample size, mean and variance (Additional file 1). Let’s consider that a variable, such as age, is reported with a mean of 60 years with a SD of 40 in a sample of size 1,000. If one assumes that age is usually normally distributed in the population, one can use the reported mean and SD to draw N independent sample from a Normal distribution with mean 60 and SD 40. The simulated datasets may *first* be used to look at the distribution of the data with two specific questions: 1) Is this distribution possible? (e.g., it is obviously impossible to observe negative values for a variable such as serum creatinine); 2) Is the distribution compatible with the variable itself? (e.g., variable for which the measurement method has a large intrinsic variability should have a variance in agreement). *Second*, it is possible to rerun the statistical analyses in each simulated dataset using the same tests as those used by the authors and building the distribution of the *p* values.

It should be emphasized that such an approach does not aim to provide new inference but only to detect some potential inconsistencies. Moreover, variable simulation from reported means and standard deviations rely on an assumption concerning the underlying distribution. Because some variables are obviously not normally distributed, it could be of interest to compare simulations under normal distribution to simulations obtained under alternate distributions. Finally, simulating 10,000 datasets is not the same as “redoing 10,000 times the same clinical trial, with the same sample size.” In such simulations, we assume observed means and standard deviations are the true population parameters, while they are in fact the observed parameters of a random sample drawn from the underlying population.

### Illustrative example

To illustrate these methods, we selected the data from a randomized study published in 2009 [10] that was based on two groups of 25 patients (Additional file 2). We chose this paper because it is known to be fraudulent and it was retracted by the Editor-in-Chief of the journal in 2010 [11]. To perform our retrospective simulations, we selected in this paper the continuous variables, the mean and the standard deviation of which were tabulated. Assuming that the reporting was appropriate, we considered that, when such variables were expressed as mean (SD), they should be normally distributed. We then used these reported means, standard deviations and the normal distribution assumption to build N = 10,000 datasets of size *n* = 50 (25 subjects in each group). Based on these simulated datasets, we reran the statistical analysis using Student *t* tests, and plotted the distribution of the observed *p* values across the 10,000 datasets. In addition, to address the skewed distribution of some variables, we also used the reported mean and standard deviations to simulate lognormal observations, and reran the analyses using nonparametric rank-sum tests.

We identified 19 tabulated continuous variables supposed to be normally distributed and for which a mean and a standard deviation were reported.

## Results

### Variables distribution

**Figure 1:**
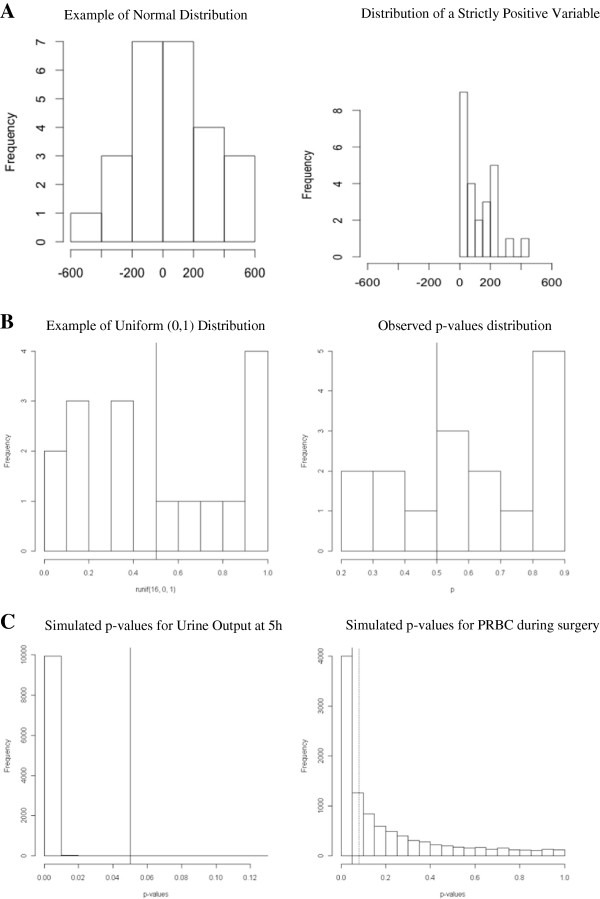
**Illustration of the checking procedure. A** Variable distribution. The variable FFP during surgery is described with a mean of 60 and a SD of 210. As shown in the left panel, if this variable was normally distributed, it should exhibit some negative values. Because negative values are impossible for such a variable, its distribution is necessarily asymmetric (right panel: example of a strictly positive variable characterized by a large SD). **B***P* value distribution under the null hypothesis. Left panel represents the theoretical distribution of the *p* values under the null hypothesis, that is a uniform (0,1) distribution: *p* values are equally distributed on both sides of the middle line. As shown in the right panel, the observed *p* values are likely not to be distributed uniformly. **C** Distribution of the simulated *p* values corresponding to the comparison of two variables in the two groups across the 10,000 simulated datasets. The left panel shows a very high probability for the comparison of the urine output at 5 hours to be statistically significant, whereas it was reported as nonsignificant by the authors. The left panel shows the distribution of the simulated *p* values concerning the comparison of the PRBC during surgery: the black vertical line represents the 0.05 threshold of statistical significance, and the dashed line represents the *p* value that has been explicitly computed, given the observed mean and SD in the two groups.

First, variables, such as duration of anesthesia, cardiopulmonary bypass (CPB), cross-clamp, and intubation, were presented using mean and standard deviation, whereas they are usually not normally distributed (Additional file 2: Table S1*a*). Second, the standard deviations for the volume of packed red blood cell (PBRC) and fresh frozen plasma (FFP) are far too large compared with their mean value (Additional file 2: Table S1*b*). As a volume cannot be negative, it is likely that these variables are not symmetrically distributed, so that the assumption underlying the use of parametric statistical tests might not hold (Figure 1A). Third, the variability of cytokines’ measurements reported in this paper is far lower than the one previously reported in the literature for similar study designs. This last point is the one that was detected by several readers, which led to the retraction.

### *P* values distribution

Under the null hypothesis of no intergroup difference after adequate randomization, the set of 16 *p* values presented in the Additional file 2: (Table S1*a*) should follow a Uniform distribution on (0,1). This means that, among 16 random values, on average, 8 are expected to be <0.5. In the present case, however, more *p* values were >0.5 (Figure 1B).

### Explicit computations

Based on the reported means and standard deviations in each randomized groups reported in the Table *b*, we recalculated the *p* values. Using this method, the results also were different from those reported by the authors for the two following variables:

Urine output at 5 hours: *p* < 10^-6^ (comparison reported as nonsignificant by the authors)

PRBC during surgery: *p* = 0.08 (comparison reported as statistically significant in the paper)

### Parametric bootstrap

We limited the simulations to those variables where (1) there seemed to be some clinical differences between the groups, but the authors reported no statistical difference, (2) the authors reported a statistically significant difference but such a difference did not seem to be of clinical relevance, or (3) the normal distribution assumption seemed to be violated. The results of the simulation are given in Table 1. Figure 1C exemplified two situations where the probability of finding the results the authors actually reported was low: the authors reported a nonstatistically significant difference in terms of urine output 5 hours after surgery, but the simulation found *p* > 0.05 in only 5 of the 10,000 simulated datasets; the volume of PRBC transfused during surgery was reported to be significantly higher in the Albumin group, but simulation showed that the probability to observe *p* < 0.05 was smaller than 0.4 (3,920/10,000, *p* < 0.05). Finally, to address the nonsymmetry of some variables, we also used the reported mean and standard deviations to simulate lognormal observations, and reran the analyses using nonparametric rank-sum tests. As reported in Table 1, results were very similar.

**Table 1:** **Distribution of the *p *values corresponding to the comparison of the two groups across the 10,000 simulated datasets**

	***p *< 0.05**	
**Variable**	**Normal distribution**	**Lognormal distributions**
	**distribution**	**distributions**
**Colloids 5 hr after surgery**	9,113/10,000	8,969/10,000
**Urine output 5 hr after**	9,995/10,000	9,985/10,000
**surgery**		
**PRBC volume**		
During surgery*	3,920/10,000	5,371/10,000
5 hr after surgery*	5,114/10,000	6,601/10,000
Until first POD*	5,533/10,000	7,133/10,000
Until second POD*	4,633/10,000	5,742/10,000
**FFP**		
**during surgery**	879/10,000	2,133/10,000
5 hr after surgery*	4,377/10,000	8,412/10,000
Until first POD*	5,880/10,000	9,446/10,000
Until second POD*	5,874/10,000	9,424/10,000

Number of *p* < 0.05 observed across the 10,000 simulated datasets. Simulations were performed using either normal or lognormal distributions. Comparisons were performed using non parametric Wilcoxon tests.

*Statistically significant comparison according to the authors.

## Discussion

We proposed a critical appraisal of the results of randomized control trials based on a multisteps procedure (Figure 1): evaluation of the distribution of the reported variables; evaluation of the distribution of the *p* values reported for the comparison of the baseline characteristics of the two groups; explicit computation of the *p* values and parametric bootstrap. The roadmap does not aim at diagnosing fraud or bias but rather at detecting some potential warnings that could be used by reviewers or editors as justifications to address queries on the raw data. If present, such warnings do not necessarily imply fraud but might only be related to some misuse of the statistical methods. Whether or not such misuses rely on fraud can be adjudicated by analyzing the raw data with variety of methods. Thereafter, the reluctance to share data might be considered as a further clue of poor quality study. Indeed, Wicherts et al. [17] recently reported that authors’ reluctance to share data is associated with more error in reporting of statistical results and with weaker evidence. Providing the availability of raw data, some authors have proposed to check more precisely the data distribution. For instance, looking at the kurtosis of the distribution, which is a measure of the shape of the distribution, might be of interest. Even if even an astute cheater would preserve the mean and the variance, he may be tripped up by examination of the kurtosis [6]. Another approach is to look at the reported numbers and especially at their digits. Indeed, the Benford’s law [18] stipulates that, under certain conditions, the distribution of the first digit of a variable follows a special logarithmic distribution [15]. This law might be used to check the randomness of the numbers reported in a paper [6]. Eventually, Masicampo et al. [19] focused on the prevalence of *p* values just greater than 0.05. They examined the distribution of *p* values reported in a large subset of papers from three highly regarded psychology journals. *P* values were much more common immediately less than 0.05 than would be expected based on the number of *p* values occurring in other ranges. The authors discussed potential sources of this pattern, including publication bias and researcher degrees of freedom. Those approaches could in turn be associated with those we suggested.

If absent, such warning signals do not exclude the presence of publication bias. Moreover, careful analysis might still fail to address other sources of bias, such as selective reporting (e.g., reporting of observations that have reached significance only) and publication bias (e.g., selective, faster, and more prominent publication of research findings that produced unusual, very strong, highly significant results) [17].

To illustrate our four-step procedure, we have applied it to analyze the data reported by Boldt et al. in an article that has been recently retracted for fraud [10,11]. It should be pointed out that the Editor-in-Chief decided to retract the article after several letters were sent to the journal pointing out the surprisingly small variability in cytokines measurements reported in the paper. Using this article as an example helped us to illustrate that, even though each step of our approach might be well known by statistically trained reviewers or readers, strictly applying this four-step procedure could have provided some important warning signals when apprising Boldt’s manuscript.

This series of analyses have some limitations. First, these analyses alone may not enable to discriminate between low- and high-quality data, because there is variety of sources of bias that may not be explored. However, in the context of medical diagnosis, we usually oppose screening vs. diagnostic tools. We ask a diagnostic tool to be very specific, while we essentially ask a screening tool to be sensitive. The objective is to offer to the patients a strategy based on a *first “screening” line* of exams (highly sensitive), which in case of positive results, would need to be confirmed by a *second line* of diagnostic exams (highly specific) in order to detect the potential false-positives. We think that our series of analysis should be considered as a *second line* of exams. It is definitely not sensitive enough to detect fraud or poor quality data in the whole “population” of manuscripts. The *first “screening” line* should be the Reviewer (or when the manuscript is already published, the reader’s own criticism sense) and even better, the statistical Reviewer. When dealing with potentially fraudulent data, it seems crucial to offer a multiple line screening. As stated by Haldane (“second order faking”), when data are fabricated to pass certain statistical tests, they are likely to fail on others [20]. Second, the series of analyses were illustrated by applying it to a single set of data [10]. A complete validation process would be needed in the near future by applying it, in a blinded manner, to a series of manuscripts. Third, the step based on parametric bootstrap may somehow be compared to retrospective power analysis, which is highly controversial [21].

## Conclusions

There is increasing concern that in modern research, false findings may be the majority of published research claims [4]. This can be view as the result of different types of fraud, or, more commonly, of inappropriate use of standard statistical tools. Simple evaluations of the reported data as those reported in the present note might offer some *warning signals.* Because the described methods are quite general, the presence of such *warning signals* should prompt asking for raw data, in order to appraise critically the quality and the validity of the data, using additional more specific tools, shaped to explore both distribution and reporting of a given variable [6]. For the future, we should concentrate our efforts to organize systematic publication of the raw data, in order to allow independent validation of the results [22-24]. Until systematic raw data access becomes the rule, the proposed tools could serve as a “lecture schedule” for both readers and reviewers.

### Key messages

– Poor-quality evidence and fraud are upcoming concerns in medical research

– Reporting guidelines should be strictly followed and imposed by medical journals

– Guidelines should be provided to reviewers to offer homogeneous evaluation of some reporting key points

– Similar simple screening tools should be available for the readers.

## Competing interests

The authors declare that they have no competing interests.

## Authors’ contributions

RP conceived and performed the analysis, wrote the manuscript, MRR, SC participated to manuscript writing, DJ participated to study design and to manuscript writing. All authors read and approved the final manuscript.

## Supplementary Material

Additional file 1 Formulas. Click here for file

Additional file 2 **Table S1 and 4 from the illustrative paper [**[10]**].** Click here for file
