# Supplementary material for: Do simple screening statistical tools help to detect reporting bias?
Source: Ann Intensive Care. 2013 Sep 2;3:29. doi: 10.1186/2110-5820-3-29 (PMC3847052; doi:10.1186/2110-5820-3-29)
Supplement: Additional file 1 — Formulas. [file 2110-5820-3-29-S1.docx]

*Additional file 1: Formulas*

**A.1 Variance computation**

When variance is not given in the manuscript, it is possible to calculate from the standard deviation or from the 95% confidence interval, suing the following formulas:

(1) $\mathrm{var}iance=SD^{2}$

(2) $LowerBoundary95CI=mean-1.96\times\sqrt{\mathrm{var}iance/n}$

$$UpperBoundary95CI=mean+1.96\times\sqrt{\mathrm{var}iance/n}$$

**A.2 Explicit p-value computation**

Under the null hypothesis of equality of mean, indeed, the p-value is given by

$$p-value=P\left(M_{1}-M_{2}>m_{1}-m_{2}\left|E\left(M_{1}\right)=E\left(M_{2}\right)\right.\right)$$

$$p-value=P\left(\frac{M_{1}-M_{2}}{\sqrt{\frac{\sigma_{1}^{2}}{n_{1}}+\frac{\sigma_{2}^{2}}{n_{2}}}}>\frac{m_{1}-m_{2}}{\sqrt{\frac{\sigma_{1}^{2}}{n_{1}}+\frac{\sigma_{2}^{2}}{n_{2}}}}\left|E\left(M_{1}\right)=E\left(M_{2}\right)\right.\right)$$

where M_1_-M_2_ is assumed to be normally distributed.
