# Supplementary material for: Do simple screening statistical tools help to detect reporting bias?
Source: Ann Intensive Care. 2013 Sep 2;3:29. doi: 10.1186/2110-5820-3-29 (PMC3847052; doi:10.1186/2110-5820-3-29)
Supplement: Additional file 2 — Table S1 and 4 from the illustrative paper [[10]]. [file 2110-5820-3-29-S2.docx]

*Additional file 2: Table S1 and 4 from the illustrative paper [10]*

**Table a. Patient Demographic and Perioperative Data (originally Table 1 in the paper)**

|  | **High-dose**  **HES group**  **(*n*** = **25)** | **Albumin based**  **group**  **(*n*** = **25)** | **p-values** |
| --- | --- | --- | --- |
| Demographics |  |  |  |
| Age (yr) | 69 ± 4 | 70 ±5 | 0.8 |
| Weight (kg) | 83 ± 13 | 80 ±11 | 0.9 |
| Height (cm) | 170 ±5 | 167 ±8 | 0.9 |
| Gender (F/M) | 14/11 | 12/13 | 0.7 |
| Preoperative medication |  |  |  |
| Aspirin | 14 | 15 | 0.6 |
| Beta-blockers | 15 | 16 | 0.9 |
| ACE inhibitors | 12 | 12 | - |
| Nitrates | 6 | 8 | 0.6 |
| AT1 inhibitors | 10 | 9 | 0.9 |
| Oral antidiabetics | 5 | 4 | 0.7 |
| Other antihypertonic drugs | 5 | 6 | 0.6 |
| Diuretics | 9 | 6 | 0.2 |
| Statins | 10 | 12 | 0.3 |
| Time of (min) |  |  |  |
| Anesthesia | 256 ±57 | 253 ± 50 | 0.4 |
| CPB | 72 ±25 | 73 ± 23 | 0.9 |
| Cross-clamp | 54 ± 16 | 59 ± 15 | 0.4 |
| Intubation | 400 ± 98 | 425 ± 101 | 0.5 |
| Outcome |  |  |  |
| ICU mortality (MOF) | 1 | 1 | - |

**Table b. Intravenous and CPB Prime Volume Administered for Groups 1 and 2. (originally table 4 in the paper)**

*p<0.05 different between the groups.

|  | **Surgery** | **5 h after surgery** | **Until first POD** | **Until second POD** |
| --- | --- | --- | --- | --- |
| Crystalloids (mL) |  |  |  |  |
| High-dose HES | 1070 ± 220* | 2010 ± 310* | 3220 ± 400* | 4010 ± 410* |
| Albumin | 2490 ± 340 | 3380 ± 300 | 5000 ± 360 | 5450 ± 560 |
| Colloids (mL) |  |  |  |  |
| High-dose HES | 2195 ± 280* | 2210 ± 250 | 2870 ± 260 | 3090 ± 340 |
| Albumin | 1580 ± 240 | 1980 ± 230 | 2820 ± 210 | 3110 ± 350 |
| Drainage blood loss (mL) |  |  |  |  |
| High-dose HES | 540 ± 70 | 790 ± 170* | 1070 ± 260* | 1200 ± 290* |
| Albumin | 610 ± 120 | 1130 ± 220 | 1440 ± 230 | 1520 ± 210 |
| Urine output (mL) |  |  |  |  |
| High-dose HES | 880 ± 320 | 2880 ± 400 | 4780 ± 420* | 6500 ± 730* |
| Albumin | 860 ± 300 | 2270 ± 410 | 4010 ± 410 | 5420 ± 700 |
| Blood/blood products (units) |  |  |  |  |
| PRBC (total number/group) |  |  |  |  |
| High-dose HES | 320 ± 300 (28)* | 350 ± 290 (32)* | 350 ± 290 (32)* | 380 ± 300 (36)* |
| Albumin | 520 ± 490 (46) | 580 ± 480 (56) | 600 ± 490 (60) | 600 ± 490 (60) |
| FFP (total number/group) |  |  |  |  |
| High-dose HES | 60 ±210 (8) | 60 ±210 (8)* | 60 ±210 (8)* | 60 ±210 (8)* |
| Albumin | 100 ±260 (12) | 200 ±319 (26) | 250 ±370 (32) | 250 ±370 (32) |
